# Preoperative red cell distribution width and neutrophil-to-lymphocyte ratio predict survival in patients with epithelial ovarian cancer

**DOI:** 10.1038/srep43001

**Published:** 2017-02-22

**Authors:** Zheng Li, Na Hong, Melissa Robertson, Chen Wang, Guoqian Jiang

**Affiliations:** 1 Department of Gynecologic Oncology, The Third Affiliated Hospital of Kunming Medical University (Yunnan Tumor Hospital), 519 Kunzhou Road, Kunming 650118, China; 2 Department of Health Sciences Research, Mayo Clinic, Rochester 55905, USA; 3 Institute of Medical Information, Chinese Academy of Medical Sciences, Beijing 100020, China

## Abstract

Several parameters of preoperative complete blood count (CBC) and inflammation-associated blood cell markers derived from them have been reported to correlate with prognosis in patients with epithelial ovarian cancer (EOC), but their prognostic importance and optimal cutoffs are still needed be elucidated. Clinic/pathological parameters, 5-year follow-up data and preoperative CBC parameters were obtained retrospectively in 654 EOC patients underwent primary surgery at Mayo Clinic. Cutoffs for neutrophil-to-lymphocyte ratio (NLR), platelet-to-lymphocyte ratio (PLR), and monocyte-to-lymphocyte ratio (MLR) were optimized by receiver operating characteristic (ROC) curve. Prognostic significance for overall survival (OS) and recurrence free survival (RFS) were determined by Cox proportional hazards models and Kaplan-Meier method. Associations of RDW and NLR with clinic/pathological parameters were analyzed using non-parametric tests. RDW with cutoff 14.5 and NLR with cutoff 5.25 had independent prognostic significance for OS, while combined RDW and NLR scores stratified patients into low (RDW-low and NLR-low), intermediate (RDW-high or NLR-high) and high risk (RDW-high and NLR-high) groups, especially in patients with high-grade serous ovarian cancer (HGSOC). Moreover, high NLR was associated with poor RFS as well. Elevated RDW was strongly associated with age, whereas high NLR was strongly associated with stage, preoperative CA125 level and ascites at surgery.

Epithelial ovarian cancer (EOC) is about one-tenth as common as breast cancer, but remains the most lethal gynecologic malignancy which serves as the fifth leading cause of cancer death among women in United States with 21,290 new cases and 14,180 deaths in 2015^1^. Less than 40% of women with EOC are cured^1^ due to 70% of patients are diagnosed with advanced disease (stage III or IV)^2^. Primary treatment for patients with advanced stage EOC consists of cytoreductive surgery, followed by platinum and taxane-based chemotherapy^3^. Unfortunately, the overall survival rate of women with EOC has changed little since platinum based treatment was introduced more than 30 years ago^4^. Known factors that influence prognosis of patients with EOC include age, FIGO stag, histology, grade and the result of surgical treatment^5^,^6^,^7^. Nevertheless, there are still patients with advanced-stage high-grade cancers survive longer than their contemporaries^8^,^9^. Although a serial of molecular signatures was reported to stratify survival in different cohorts of EOC patients^10^,^11^,^12^,^13^, simple, reproducible and inexpensive biomarkers to generate prognostic model are still unavailable at clinical settings.

Complete blood count (CBC) is one of the most simple, reproducible and inexpensive tests for patients with EOC. In addition to guiding the clinical management of EOC patients who are candidates for surgery, parameters of preoperative CBC, such as platelet count^14^, hemoglobin^15^,^16^, and eosinophil count^17^, were also reported to correlate with survival of patients. Moreover, with accumulating evidences on the role of inflammation in carcinogenesis and tumor progression^18^,^19^, several serum parameters as markers of systemic inflammation, ranging from neutrophil-to-lymphocyte ratio (NLR) and platelet-to-lymphocyte ratio (PLR), to monocyte-to-lymphocyte ratio (MLR), have been revealed to possess potential to predict survival in a variety of human cancers^20^,^21^,^22^,^23^, including EOC^24^,^25^,^26^,^27^. Quite recently, some other inflammation-associated blood cell markers, such as red cell distribution width (RDW), have also been shown to associate with survival of solid tumors^28^,^29^, but have never been studied in EOC. Thus, the aim of the current study was to investigate simultaneously the impact of preoperative CBC parameters and inflammation-associated blood cell markers on survival of a large cohort of EOC patients with 5-year follow-up data.

## Results

### Patient characteristics

Patient characteristics are outlined in Table 1. A total of 654 patients with EOC were included in the present study. Median age at diagnosis was 63 years (range 28–93). Most patients (533, 81.5%) was of advanced stage (stage III or IV) and underwent cytoreductive surgery, followed by platinum and taxane-based chemotherapy. There were 482 (73.7%) patients with cancer originated from ovary (excluded fallopian tube cancer and primary peritoneal cancer) and among them, 355 (73.7%) were with high-grade serous ovarian cancer (HGSOC), the most common and lethal subtypes of EOC^30^. Median follow-up time for the current cohort was 49.5 months (range 0.1–175.3).

### Prognostic significance of preoperative CBC parameters and cut-off determination

To elucidate the prognostic significance of preoperative CBC parameters and inflammation-associated blood cell markers, univariate Cox proportional hazards analyses were performed on continuous data (Supplementary Table 1). Platelet, leukocyte, erythrocyte, neutrophils and lymphocyte counts, along with hemoglobin and hematocrit are significantly associated with OS, while platelet, neutrophils and lymphocyte counts are associated with RFS. Analyses also revealed elevated RDW, PLR, NLR and MLR are associated with both poor OS and RFS. However, no association between monocyte, basophil or eosinophil counts and survival were found.

Cutoff values for PLR applied to predict survival in EOC patients range from 200^26^,^27^ to 300^31^, while those for NLR vary from 2.6^24^ to 3.77^32^, or even a trend rather than specific values^25^ that cripples their prognostic values for clinical use. Moreover, there is no report concerning the prognostic significance, yet the cutoff values for RDW and MLR in EOC until now. Thus, we decide to optimize cutoff for RDW, PLR, NLR and MLR on this study cohort with ROC curve analysis (Materials and Methods). Cutoff values as 14.15 for RDW (P = 5.6^e-4^, HR = 141), 5.25 for NLR (P = 1^e-4^, HR = 1.48), 273.5 for PLR (P = 5^e-8^, HR = 1.68) and 0.45 for MLR (P = 8.5^e-8^, HR = 1.66) were then optimized respectively.

### RDW and NLR have independent prognostic significance

Univariate Cox proportional hazards analyses also revealed age at diagnosis (stratified into four groups according to interquartile range), origin of cancer (EOC, fallopian tube cancer (FTC), and primary peritoneal cancer (PPC)), stage, histology, grade, preoperative CA125 level (≥35 vs <35 U/ml, P < 0.001), ascites at surgery (yes vs no, P < 0.001) and residual disease were significantly associated with OS (Table 2), while all except age were significantly associated with RFS (Supplemental Table 2). We then included those clinical/pathological parameters except preoperative CA125 and ascites at surgery because of a large amount of missing values, into subsequent multivariate Cox proportional hazards models.

**Figure 1:**
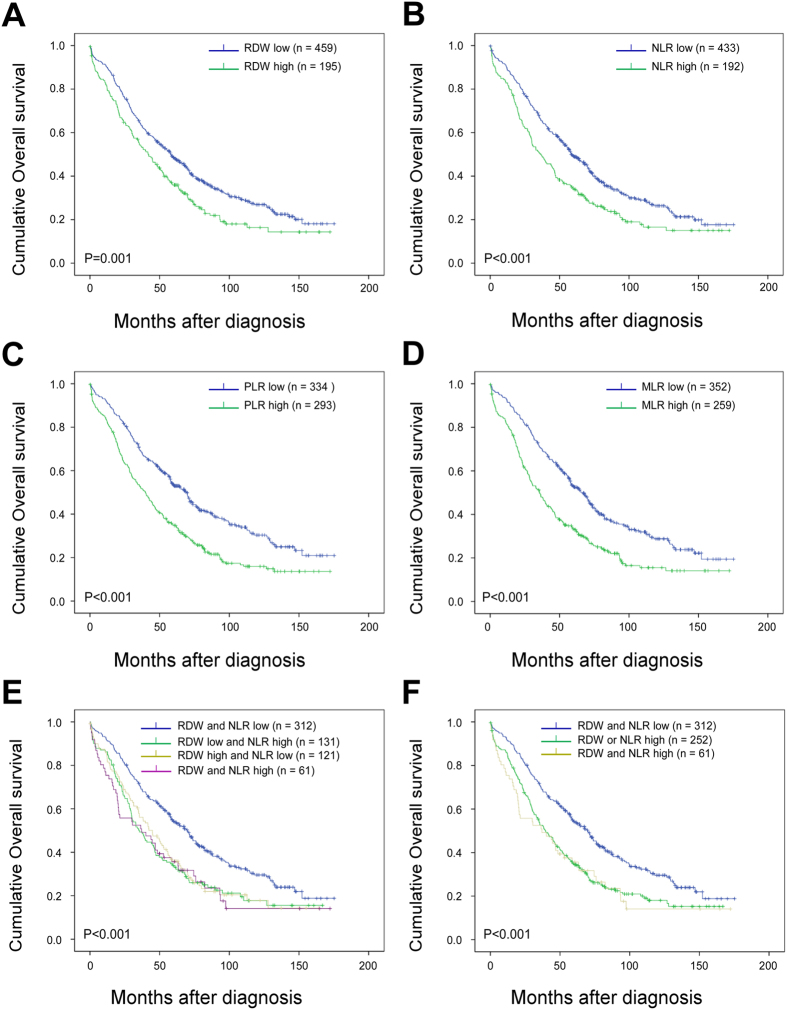
Overall survival of ovarian cancer patients stratified according to RDW, NLR and MLR cut-offs (N = 654). Kaplan–Meier overall survival (OS) curves with log-rank P-values for patients stratified using red blood cell distribution width (RDW) cutoff of 14.15 (**A**), neutrophil-to-lymphocyte ratio (NLR) cutoff of 5.25 (**B**), platelet-to-lymphocyte ratio (PLR) cutoff of 242.9 (**C**), monocyte-to-lymphocyte ratio (MLR) cutoff of 0.45 (**D**) and combined RDW + NLR (four groups, **E**; three groups, **F**) with cutoffs defined above.

**Figure 2:**
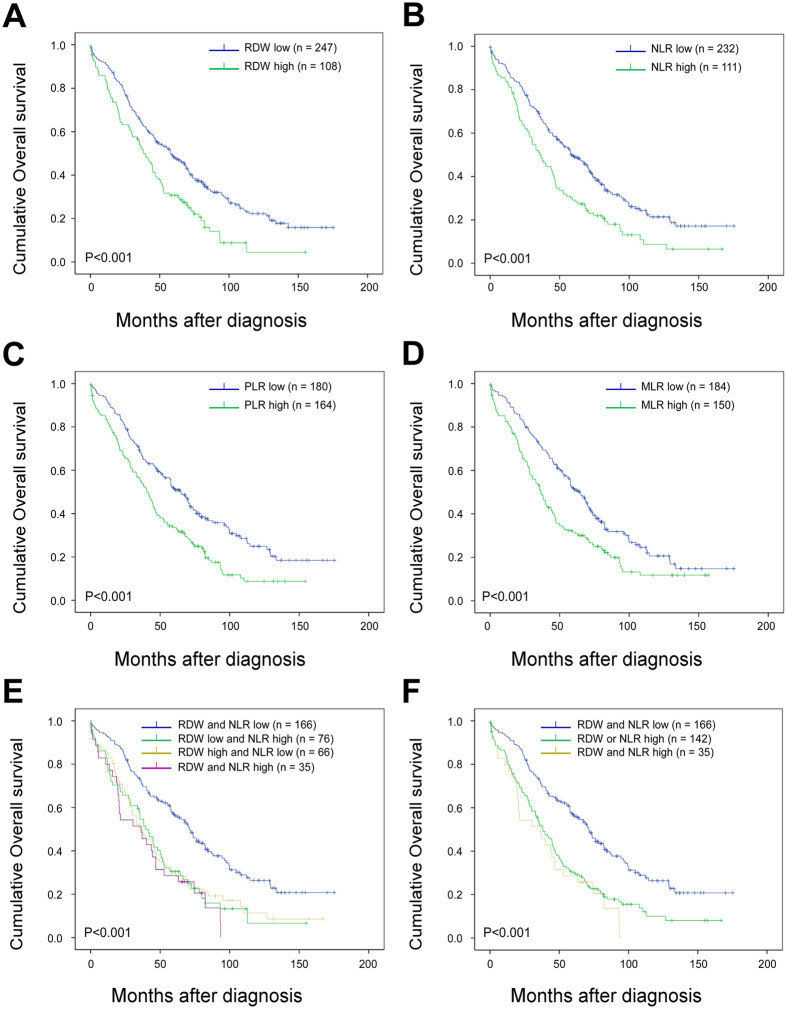
Overall survival of high-grade serous ovarian cancer patients stratified according to RDW, NLR and MLR cut-offs (N = 355). Kaplan–Meier overall survival (OS) curves with log-rank P-values for patients stratified using red blood cell distribution width (RDW) cutoff of 14.15 (**A**), neutrophil-to-lymphocyte ratio (NLR) cutoff of 5.25 (**B**), platelet-to-lymphocyte ratio (PLR) cutoff of 242.9 (**C**), monocyte-to-lymphocyte ratio (MLR) cutoff of 0.45 (**D**) and combined RDW+NLR (four groups, **E**; three groups, **F**) with cutoffs defined above.

Kaplan-Meier analysis and log-rank test demonstrated that high preoperative RDW, NLR, PLR and MLR significantly predicted poorer OS both in all EOC patients (including FTC and PPC, Fig. 1A to D) and in HGSOC patients (Fig. 2A to D). Patients with different combination of RDW and NLR according to their dichotomized values had extra prognostic values both in all EOC patients (including FTC and PPC, Fig. 1E) and in HGSOC patients (Fig. 2E). We then combined RDW+NLR by stratifying patients into three, rather than four groups for more distinctive patients’ stratification and easier clinical usage^33^,^34^ as: (1) RDW-low and NLR-low; (2) RDW-high or NLR-high; and (3) RDW-high and NLR-high. The simplest and most effective way succeeded in identifying low, intermediate and high risk groups, especially in HGSOC patients, with estimated cumulative 5-year OS rates of 58.4%, 31.4% and 24.4%, respectively (Fig. 2F), as well as 53.8%, 34.5% and 35.7%, respectively, in all EOC patients (Fig. 1F). So, we used three groups strategy rather than four groups to summarize combined RDW+NLR in the following analyses.

Given that NLR, PLR and MLR were strongly correlated with each other (Spearman’s rho coefficients of 0.425 (NLR vs PLR), 0.511 (NLR vs MLR) and 0.514 (PLR vs MLR; all P < 0.001), and all of them were derived from CBC parameters such as platelet, neutrophils, monocyte and lymphocyte counts, all CBC parameters and inflammation-associated blood cell markers that had significantly impact on survival in univariate Cox proportional hazards analyses (P < 0.05) were adjusted separately in multivariable Cox proportional hazards models included (1) age at diagnosis, origin of cancer, stage, histology, grade and residual disease for OS in all EOC patients (Table 2); (2) age at diagnosis, stage and residual disease for OS in HGSOC patients (Table 3); and (3) origin of cancer, stage, histology, grade and residual disease for RFS in all EOC patients (Supplemental Table 2). RDW, NLR and combined RDW+NLR were then revealed as independent prognostic factors for OS both in all EOC patients and in HGSOC patients, while more highly significant in HGSOC patients (except for RDW in HGSOC patients, P = 0.064). However, no association between other CBC parameters and inflammation-associated blood cell markers with OS was identified by multivariate Cox proportional hazards analyses. On the contrary, only NLR had independent prognostic value for RFS both in all EOC patients (Supplemental Fig. 1A) and in HGSOC patients (Supplemental Fig. 1B).

### Associations of RDW and NLR with other clinic/pathological parameters

Finally, associations of RDW and NLR with other clinic/pathological parameters were investigated (Table 4). While RDW was significantly associated with age (<0.001) and significantly elevated in patients aged ≥72 years compared with those aged <55 years, NLR was significantly associated with features of high tumor burden, such as stage (P = 0.006), preoperative CA125 level (P = 0.001) and ascites at surgery (P < 0.001).

## Discussion

Here is the first study to investigate the prognostic value of preoperative RDW in EOC, and the largest study to investigate the prognostic role of preoperative CBC parameters and inflammation-associated blood cell markers including NLR, PLR and MLR in patients with EOC, especially in patients with HGSOC. We revealed that elevated preoperative RDW and NLR predict poor OS in patients with EOC, and combined high RDW+NLR provides additional patient stratification, especially in patients with HGSOC. On the contrary, only high NLR predicts poor RFS in EOC and HGSOC.

Inflammation has been recognized as one of the hallmarks of nearly all human cancers^35^. Tumor-related inflammatory microenvironment could facilitate tumor growth and metastasis by sustaining proliferation, inhibiting apoptosis, inducing epithelial-to-mesenchymal transition (EMT), initiating angiogenesis, and suppressing host-anti-tumor immunity^18^,^19^. For EOC, epidemiological studies revealed pelvic inflammatory diseases might increase risk^36^, while inflammation caused by incessant ovulation remains one of the well-accepted hypotheses of EOC carcinogenesis^37^. However, the biologic mechanisms underlying the correlation between NLR, PLR, MLR, systemic inflammation and tumorigenesis in EOC remains poorly understood. In fact, intratumoral neutrophils had been shown to be associated with unfavorable survival in many human cancers ranging from hepatocellular carcinoma, gastric carcinoma, colorectal cancer, to non-small-cell lung cancer and renal cell carcinoma^38^. Cancer cells facilitate recruitment of tumor-associated neutrophils (TANs) by expressing various of chemokines and cytokines, including CXCL5, CXCL6, and CXCL8^39^, along with ligands that recognize receptors such as CXCR2 expressed by TANs^40^. TANs recruited by tumor then release pro-growth and pro-invasion factors including hepatocyte growth factor (HGF), reactive oxygen species (ROS), reactive nitrogen species (RNS), neutrophil elastase (NE), neutrophil collagenase (MMP8), and gelatinase B (MMP9). In addition, TANs also release cytokines like Oncostatin M, which induce VEGF and then stimulate angiogenesis to support tumor metastasis^41^. On the contrary, lymphocytes, especially CD8+T cells, which represents host anti- tumor immune response, had been recognized as a predictor of favorable survival in a variety of human cancers^42^, including EOC^43^. However, neutrophils recruited by tumor could interact with CD8+T cells to counteract their protective effect that result in procancer immunosuppressive microenvironment^41^. That may explain, why high preoperative NLR, in terms of more neutrophils and less lymphocytes, was significantly associated with features of high tumor burden, including stage (P = 0.006), preoperative CA125 level (P = 0.001) and ascites at surgery (P < 0.001), and predicted both poor RFS and OS in EOC patients in the current study. Quite recently, two independent studies conducted in colorectal cancer added evidence to the hypothesis mentioned above. Chen, Z. Y. *et al*. indicated NLR > 5 was associated with poor prognosis in metastatic colorectal cancer and high NLR was correlated with increased expression of inflammatory cytokines such as interleukin 6 (IL-6), IL-8, IL-2Ra, HGF, macrophage-colony stimulating factor (M-CSF), and vascular epidermal growth factor (VEGF)^44^. Pine, J. K. and colleagues found NLR ≥ 5 predicted lower overall survival and greater disease recurrence while lower NLR was associated with pronounced lymphocytic reaction at the invasive margin (IM) in colorectal cancer tissues^45^. Those results inspired us to further study the cytokines profile and tumor associated local lymphocytic response in this EOC cohort to better understand the possible mechanism behind preoperative high NLR as a risk factor predicting poor prognosis in EOC patients.

Previous studies in EOC^26^,^27^,^31^,^46^ and many other human cancers^22^ established NLR’s role in predicting survival, but the wide range of NLR cutoff from 1.9 to 5.0^20^ limited its usage in clinical field. This study employed ROC curve analysis to optimize cutoff for NLR as 5.25, which succeeded in stratifying 654 EOC patients independently into two distinctive survival groups both for RFS (P = 0.026, HR = 1.331, 95% CI = 1.035–1.712, multivariate) and OS (P = 0.002, HR = 1.391, 95% CI = 1.133–1.708). While studies in independent cohort to determine the optimized cutoffs for NLR in EOC are still warranted.

Also known as erythrocytes, red blood cells (RBCs) are the most common type of blood cells^47^. Red cell distribution width (RDW) indicates the size variation of RBCs, and is calculated by dividing the mean corpuscular volume (MCV) by the standard deviation (SD) of the RBC and then multiplied for 100, to express data as a percentage^48^. Traditionally, RDW is used in laboratory hematology for differential diagnosis of anemias^49^, while quite recently, growing evidence indicated that high RDW is associated with systematic inflammation^49^ and elevated RDW harbored the potential to predict poor survival in a variety of human cancers, consisting of breast cancer^50^,^51^,^52^, lung cancer^53^,^54^,^55^, prostate cancer^56^, endometrial cancer^57^ and upper tract urothelial carcinoma^58^. In the present study, we demonstrated, for the first time, that preoperative RDW, with cutoff 14.15 determined by ROC curve, acts as a risk factor for shorten OS in patients with EOC. Moreover, combined high RDW+NLR provides additional patient stratification, especially in patients with HGSOC (estimated cumulative 5-year OS rates of 58.4%, 31.4% and 24.4%, respectively), even though RDW itself lost impact on OS in multivariate model in HGSOC patients (P = 0.064). Given that 81.5% patients underwent adjuvant platinum and taxane-based chemotherapy after surgery, these data suggest that patients with high preoperative RDW and NLR might be potential candidates for clinical trials employing more intensive treatments, including maintain chemotherapy, target therapy and immune therapy to delay recurrence and obtain desirable prognosis. However, RDW’s significant association with age (<0.001) in this EOC cohort indicated that RDW may serve as a surrogate for conditions like poor performance/nutrition status and the presence of comorbidities, which needs to be elucidated in studies involving cancer-specific death analyses.

In conclusion, the current study highlights the role of RDW and NLR as additional prognostic factors in EOC patients. These simple, reproducible and inexpensive markers, though need further investigations, may harbor the potential to identify high-risk EOC patients as candidate for more intensive therapies after standard treatment.

## Material and Methods

### Patients and follow-up

Patients who underwent primary surgery for invasive EOC, fallopian tube cancer (FTC), or primary peritoneal cancer (PPC) from 2000 to 2010 at departments of gynecologic surgery at Mayo Clinic in Rochester, MN were recruited. FTC and PPC are less common neoplasms that are managed in a similar manner to epithelial ovarian cancer^3^. The research was approved by the institutional review board (IRB) of Mayo Clinic. All methods were performed in accordance with the relevant guidelines and regulations. Patients provided written informed consent and permission for active follow-up concerning of recurrence and vital status changes. Patients were excluded if they (1) underwent neoadjuvant chemotherapy prior to surgery; (2) underwent prior surgery for their cancer elsewhere; (3) were treated as recurrent disease; (4) had non-epithelial or non-ovarian malignancies; (5) had no preoperative CBC parameters tested by Mayo Clinic in Rochester within 30 days prior to primary surgery or (6) did not consent to the use of their medical records for research purposes. Perioperative CBC parameters were collected retrospectively. Patient cohort identification and data query were supported by our data management and analysis platform named Integrating Biology and the Bedside (i2b2)^59^, an NIH-funded software framework allowing collaborative exchange of data including electronic health records, lab results, genetic and research data. Details of cohort identification and data query will be described in our other publications. Recurrence and vital status were updated every six months using medical records and active follow-up. The end of follow-up was the time of last follow-up (April 2015) or death.

### Statistical analysis

Overall survival (OS) was defined as time from diagnosis to death (all causes). Recurrence free survival (RFS) was defined as time from surgery to the first recurrence, and patients who had persistent disease after primary treatment (surgery alone or surgery and adjuvant chemotherapy) were treated as censored. Cutoff optimization of RDW, NLR, PLR and MLR were performed using the software package Cutoff Finder^33^ based on R version 2.15.0 (R Core Team, 2012), and the standard receiver operating characteristic (ROC) curve based on binary outcome (vital status), using Manhattan distance to calculate optimal cut-offs was employed. Univariate and multivariate survival analyses were performed using Cox proportional hazards models. Kaplan-Meier method and corresponding log rank test were used for survival analyses on categorical variables. Correlations between RDW, NLR, PLR and MLR were performed using Spearman’s rho test. Associations of RDW and NLR with other categorized clinic/pathological parameters were determined using either Mann-Whitney U-tests or Kruskal-Wallis tests followed by post-hoc pairwise Mann-Whitney U-tests.

All statistical tests were two-sided, and P-values < 0.05 were considered significant. Statistical analysis was performed using IBM SPSS package (Statistical Package for the Social Sciences; Version 22, Armonk, NY).

## Additional Information

**How to cite this article:** Li, Z. *et al*. Preoperative red cell distribution width and neutrophil-to-lymphocyte ratio predict survival in patients with epithelial ovarian cancer. *Sci. Rep.* **7**, 43001; doi: 10.1038/srep43001 (2017).

**Publisher's note:** Springer Nature remains neutral with regard to jurisdictional claims in published maps and institutional affiliations.

## Supplementary Material

Supplementary Dataset

## Figures and Tables

**Table 1:** Patient characteristics (N = 654).

Covariates	No. of Patients (%)
Age at diagnosis, years	
Mean, Range	63.0, 28–93
Race	
White	563 (97.2%)
Asian	3 (0.5%)
Other	13 (2.2%)
Origin of cancer	
Ovary	482 (73.7%)
Fallopian tube	10 (1.5%)
Peritoneum	162 (24.8%)
Stage	
I	87 (13.3%)
II	34 (5.2%)
III	416 (63.6%)
IV	117 (17.9%)
Histology	
High-grade serous	525 (80.3%)
Low-grade serous	4 (0.6%)
Endometrioid	71 (10.9%)
Clear cell	37 (5.7%)
Mucinous	17 (2.6%)
Grade	
1	28 (4.3%)
2	54 (8.3%)
3	572 (87.5%)
Preoperative CA125 level, U/ml	
<35	50 (9.5%)
≥35	475 (90.5%)
Unknown	129
Ascites at surgery	
No	187 (34.2%)
Yes	359 (65.8%)
Unknown	108
Residual disease	
None	266 (41.0%)
Macroscopic disease <1 cm	305 (47.1%)
Macroscopic disease >1 cm	77 (11.9%)
Recurrence	
No	276 (42.2%)
Yes	293 (44.8%)
Unknown	85
Vital status	
Alive	197 (30.1%)
Dead	457 (69.9%)

Numbers may not add to total due to missing values (75 for race, 6 for surgical debulking).

**Table 2:** Overall survival of ovarian cancer patients stratified according to RDW, NLR, PLR and MLR cut-offs, together with other prognostic parameters (N = 654).

Parameter	Univariate		Multivariate	
	HR (95% CI)	*P*	HR (95% CI)	*P*
RDW				
Low (<14.15)	1 (reference)		1 (reference)	
High (≥14.15)	1.412 (1.160–1.720)	0.001	1.235 (1.008–1.513)	0.042
NLR				
Low (<5.25)	1 (reference)		1 (reference)	
High (≥5.25)	1.478 (1.212–1.801)	<0.001	1.391 (1.133–1.708)	0.002
PLR				
Low (<273.5)	1 (reference)		1 (reference)	
High (≥273.5)	1.680 (1.392–2.028)	<0.001	1.102 (0.900–1.348)	0.347
MLR				
Low (<0.45)	1 (reference)		1 (reference)	
High (≥0.45)	1.565 (1.412–20.69)	<0.001	1.129 (0.923–1.381)	0.236
Combined RDW+NLR				
RDW-low + NLR-low	1 (reference)		1 (reference)	
RDW-high or NLR-high	1.595 (1.307–1.946)	<0.001	1.332 (1.087–1.633)	0.006
RDW-high + NLR-high	1.734 (1.261–2.385)	0.001	1.670 (1.207–2.311)	0.002
Age at diagnosis, years				
<55	1 (reference)		**1 (reference)**	
55–63	1.351 (1.019–1.790)	0.036	**1.032 (0.767–1.389)**	**0.834**
63–72	1.762 (1.356–2.290)	<0.001	**1.216 (0.925–1.597)**	**0.161**
≥72	2.539 (1.955–3.296)	<0.001	**1.948 (1.476–2.571)**	**<0.001**
Origin of cancer				
Ovary	1 (reference)		**1 (reference)**	
Fallopian tube	0.790 (0.352–1.774)	0.568	**0.962 (0.424–2.182)**	**0.925**
Peritoneum	1.872 (1.531–2.289)	<0.001	**1.218 (0.985–1.507)**	**0.069**
Stage				
I	1 (reference)		**1 (reference)**	
II	1.752 (0.871–3.522)	0.116	**1.260 (0.598–2.654)**	**0.543**
III	5.980 (3.799–9.415)	<0.001	**2.647 (1.486–4.714)**	**0.001**
IV	9.971 (6.161–16.136)	<0.001	**4.201 (2.281–7.736)**	<**0.001**
Histology				
High-grade serous	1 (reference)		**1 (reference)**	
Low-grade serous	0.175 (0.025–1.247)	0.082	**0.344 (0.036–3.262)**	**0.353**
Endometrioid	0.338 (0.232–0.494)	<0.001	**1.039 (0.653–1.655)**	**0.870**
Clear cell	0.497 (0.306–0.808)	0.005	**1.260 (0.706–2.248)**	**0.435**
Mucinous	0.082 (0.020–0.330)	<0.001	**0.369 (0.085–1.846)**	**0.238**
Grade				
1	1 (reference)		**1 (reference)**	
2	1.667 (0.662–4.200)	0.278	**0.607 (0.201–1.839)**	**0.378**
3	6.592 (2.940–14.781)	<0.001	**1.249 (0.422–3.703)**	**0.688**
Residual disease				
None	1 (reference)		**1 (reference)**	
Macroscopic disease <1 cm	2.981 (2.399–3.705)	<0.001	**1.823 (1.432–2.320)**	<**0.001**
Macroscopic disease >1 cm	5.427 (4.037–7.296)	<0.001	**2.939 (2.123–4.069)**	<**0.001**

Univariate and multivariate analysis performed using Cox proportional hazards models. RDW, NLR, PLR, MLR and combined RDW+NLR were adjusted separately in models that included age at diagnosis, origin of cancer, stage, histology, grade and residual disease. Preoperative CA125 level and ascites at surgery were excluded because of missing values (19.7% and 16.5%, respectively). Results from multivariate model which included combined RDW+NLR score are indicated in bold. Abbreviations: RDW = red blood cell distribution width; NLR = neutrophil-to-lymphocyte ratio; PLR = platelet-to-lymphocyte ratio; MLR = monocyte-to-lymphocyte ratio; HR = hazard ratio; CI = confidence interval.

**Table 3:** Overall survival of high-grade serous ovarian cancer patients stratified according to RDW, NLR, PLR and MLR cut-offs, together with other prognostic parameters (N = 355).

Parameter	Univariate		Multivariate	
	HR (95% CI)	*P*	HR (95% CI)	*P*
RDW				
Low (<14.15)	1 (reference)		1 (reference)	
High (≥14.15)	1.682 (1.299–2.180)	<0.001	1.292 (0.985–1.694)	0.064
NLR				
Low (<5.25)	1 (reference)		1 (reference)	
High (≥5.25)	1.663 (1.287–2.149)	<0.001	1.414 (1.087–1.838)	0.010
PLR				
Low (<273.5)	1 (reference)		1 (reference)	
High (≥273.5)	1.683 (1.313–2.158)	<0.001	1.228 (0.935–1.613)	0.139
MLR				
Low (<0.45)	1 (reference)		1 (reference)	
High (≥0.45)	1.600 (1.245–2.057)	<0.001	1.156 (0.883–1.514)	0.291
Combined RDW+NLR				
RDW-low+NLR-low	1 (reference)		1 (reference)	
RDW-high or NLR-high	1.900 (1.458–2.477)	<0.001	1.392 (1.058–1.830)	0.018
RDW-high+NLR-high	2.342 (1.553–3.530)	<0.001	1.844 (1.213–2.804)	0.004
Age at diagnosis, years				
<55	1 (reference)		**1 (reference)**	
55–63	1.351 (1.019–1.790)	0.036	**1.099 (0.749–1.610)**	**0.630**
63–72	1.762 (1.356–2.290)	<0.001	**1.232 (0.866–1.753)**	**0.246**
≥72	2.539 (1.955–3.296)	<0.001	**2.233 (1.566–3.184)**	<**0.001**
Stage				
I	1 (reference)		**1 (reference)**	
II	1.752 (0.871–3.522)	0.116	**1.597 (0.574–4.444)**	**0.370**
III	5.980 (3.799–9.415)	<0.001	**2.452 (1.050–5.725)**	**0.038**
IV	9.971 (6.161–16.136)	<0.001	**4.649 (1.908–11.327)**	**0.001**
Residual disease				
None	1 (reference)		**1 (reference)**	
Macroscopic disease <1 cm	2.981 (2.399–3.705)	<0.001	**1.491 (1.094–2.032)**	**0.011**
Macroscopic disease >1 cm	5.427 (4.037–7.296)	<0.001	**2.908 (1.929–4.384)**	<**0.001**

Univariate and multivariate analysis performed using Cox proportional hazards models. RDW, NLR, PLR, MLR, and combined RDW+NLR were adjusted separately in models that included age at diagnosis, stage and residual disease. Preoperative CA125 level and ascites at surgery were excluded because of missing values (16.3% and 18.3%, respectively). Results from multivariate model which included combined RDW+NLR score are indicated in bold. Abbreviations: RDW = red blood cell distribution width; NLR = neutrophil-to-lymphocyte ratio; PLR = platelet-to-lymphocyte ratio; MLR = monocyte-to-lymphocyte ratio; HR = hazard ratio; CI = confidence interval.

**Table 4:** Associations of RDW and NLR with other clinic/pathological parameters (N = 654).

Parameter	n%^#^	RDW, Median (IQR)	*P*	NLR, Median (IQR)	*P*
Age at diagnosis, years					
<55	180 (27.5)	13.70 (12.60–14.10)^4^	<0.001	5.13 (2.83–6.06)	0.210
55–63	151 (23.1)	13.71 (12.70–14.40)		5.04 (3.09–5.71)	
63–72	171 (26.1)	13.83 (12.80–14.30)		4.71 (2.88–5.64)	
≥72	152 (23.2)	14.16 (13.10–14.80)^1^		5.13 (2.96–5.21)	
Origin of cancer					
Ovary	482 (73.7)	13.88 (12.80–14.50)	0.576	4.94 (2.94–5.67)	0.359
Fallopian tube	10 (1.5)	14.17 (12.60–15.83)		3.34 (2.11–4.74)	
Peritoneum	162 (24.8)	13.72 (12.80–14.30)		4.87 (3.19–5.67)	
Stage					
I	87 (13.3)	14.00 (12.90–14.10)	0.441	4.07 (2.55–5.20)^4^	0.006
II	34 (5.2)	13.41 (12.65–14.33)		3.64 (2.49–4.20)^3,4^	
III	416 (63.6)	13.82 (12.70–14.50)		4.86 (2.94–5.70)^2,4^	
IV	117 (17.9)	13.94 (12.90–14.60)		5.96 (3.70–6.22)^1,2,3^	
Histology					
High-grade serous	525 (80.3)	13.80 (12.75–14.40)	0.499	4.99 (2.97–5.66)	0.378
Low-grade serous	4 (0.6)	14.80 (13.60–14.70)		2.95 (2.55-NA)	
Endometrioid	71 (10.9)	14.06 (12.90–14.50)		4.33 (2.62–5.74)	
Clear cell	37 (5.7)	14.06 (12.90–14.80)		5.14 (3.13–6.43)	
Mucinous	17 (2.6)	13.53 (12.75–14.25)		3.86 (2.99–4.94)	
Grade					
1	28 (4.3)	14.41 (12.93–14.75)	0.437	3.82 (2.55–4.94)	0.149
2	54 (8.3)	13.51 (12.80–14.10)		3.79 (2.32–4.85)	
3	572 (87.5)	13.85 (12.80–14.50)		5.04 (3.04–5.70)	
Preoperative CA125 level, U/ml					
<35	50 (7.6)	13.98 (12.95–14.25)	0.788*	3.23 (1.92–4.41)	0.001*
≥35	475 (72.6)	13.77 (12.70–14.30)		5.09 (3.11–5.72)	
Missing	129 (19.7)	—		—	
Ascites at surgery					
No	187 (28.6)	13.71 (12.80–14.00)	0.004*	4.10 (2.56–5.10)	<0.001*
Yes	359 (54.9)	13.91 (12.80–14.60)		5.44 (3.31–6.27)	
Missing	108 (16.5)	—		—	
Residual disease					
None	266 (40.7)	13.75 (12.80–14.10)	0.234	4.26 (2.61–5.45)	0.348
Macroscopic disease <1 cm	305 (46.6)	13.85 (12.80–14.40)		5.09 (3.06–5.74)	
Macroscopic disease >1 cm	77 (11.8)	14.01 (12.70–14.95)		6.45 (3.80–6.97)	
Missing	6 (0.9)	—		—	

^#^Values for RDW. *P-values from Mann–Whitney U-test (all other P-values are from Kruskall–Wallis tests). 1,2,3,4Categories significant differences between one another following post-hoc Mann–Whitney U-tests with Bonferroni corrections for multiple comparisons. Abbreviations: RDW = red blood cell distribution width; NLR = neutrophil-to-lymphocyte ratio; NA = no data available due to small sample size in that category.
