# Supplementary material for: Preoperative red cell distribution width and neutrophil-to-lymphocyte ratio predict survival in patients with epithelial ovarian cancer
Source: Sci Rep. 2017 Feb 22;7:43001. doi: 10.1038/srep43001 (PMC5320446; doi:10.1038/srep43001)
Supplement: Supplementary Dataset [file srep43001-s1.doc]

# Preoperative red cell distribution width and neutrophil-to-lymphocyte ratio predict survival in patients with epithelial ovarian cancer

Zheng Li^1, 2*+^, Na Hong^2, 3+^, Melissa Robertson^2^, Chen Wang^2^, & Guoqian Jiang^2*^

^1^Department of Gynecologic Oncology, The Third Affiliated Hospital of Kunming Medical University (Yunnan Tumor Hospital), 519 Kunzhou Road, Kunming 650118, China.

^2^Department of Health Sciences Research, Mayo Clinic, Rochester 55905, USA.

^3^Institute of Medical Information, Chinese Academy of Medical Sciences, Beijing 100020 China.


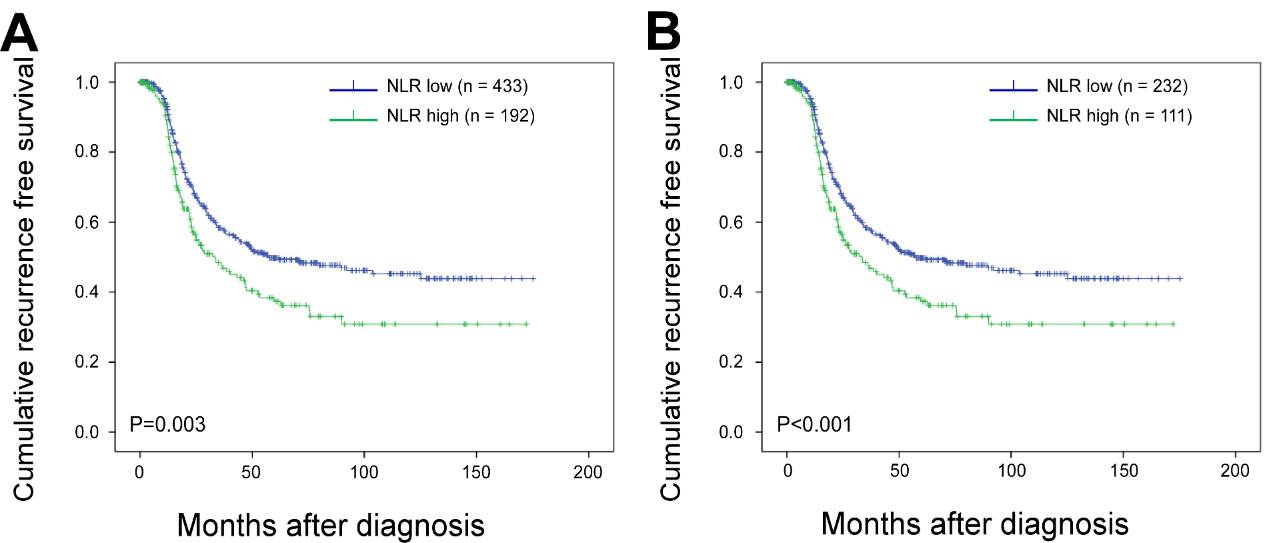


**Supplemental Figure 1.** Recurrence free survival of ovarian cancer patients stratified according to NLR cut-off. Kaplan–Meier recurrence free survival (RFS) curves with log-rank P-values for patients stratified using neutrophil-to-lymphocyte ratio (NLR) cut-off of 5.25 for ovarian cancer patients with all histologic types (N=654, A), and high-grade serous ovarian cancer patients (N=355, B).

Supplementary Table 1. Univariate survival analysis of preoperative blood parameters as continuous variables

|  | **Overall Survival** | | **Recurrence Free Survival** | |
| --- | --- | --- | --- | --- |
| **Parameter** | **HR (95% CI)** | ***P*** | **HR (95% CI)** | ***P*** |
| Platelets | 1.001 (1.001 - 1.002) | <0.001 | 1.001 (1.000 - 1.002) | 0.001 |
| Hemoglobin | 0.898 (0.841 - 0.959) | 0.001 | 0.984 (0.906 - 1.069) | 0.705 |
| Leukocytes | 1.035 (1.001 - 1.069) | 0.041 | 1.021 (0.979 - 1.066) | 0.331 |
| Hematocrit | 0.966 (0.944 - 0.989) | 0.004 | 0.996 (0.967 - 1.025) | 0.774 |
| MCV | 0.994 (0.978 - 1.011) | 0.500 | 0.983 (0.964 - 1.003) | 0.088 |
| RDW | 1.058 (1.009 - 1.109) | 0.020 | 0.973 (0.906 - 1.046) | 0.462 |
| Erythrocytes | 0.777 (0.634 - 0.952) | 0.015 | 1.120 (0.870 - 1.442) | 0.380 |
| Neutrophils | 1.051 (1.016 - 1.089) | 0.005 | 1.049 (1.003 - 1.096) | 0.038 |
| Lymphocytes | 0.718 (0.594 - 0.868) | 0.001 | 0.724 (0.573 - 0.916) | 0.007 |
| Monocytes | 1.193 (0.982 - 1.448) | 0.075 | 1.106 (0.847 - 1.444) | 0.460 |
| Basophils | 1.036 (0.962 - 1.116) | 0.353 | 1.060 (0.967 - 1.161) | 0. 213 |
| Eosinophils | 0.994 (0.938 - 1.054) | 0.852 | 0.935 (0.862 - 1.014) | 0. 104 |
| PLR | 1.001 (1.001 - 1.001) | <0.001 | 1.001 (1.000 - 1.001) | <0.001 |
| NLR | 1.038 (1.017 - 1.059) | <0.001 | 1.037 (1.011 - 1.063) | 0.005 |
| MLR | 1.941 (1.496 - 2.518) | <0.001 | 1.922 (1.348 - 2.740) | <0.001 |

Univariate analysis performed using Cox proportional hazards models. Abbreviations: MCV= mean corpuscular volume; RDW = red blood cell distribution width; NLR = neutrophil-to-lymphocyte ratio; PLR = platelet-to-lymphocyte ratio; MLR = monocyte-to-lymphocyte ratio; HR = hazard ratio; CI = confidence interval.

**Supplementary Table 2. Recurrence free survival of ovarian cancer patients stratified according to RDW, NLR. PLR and MLR cut-offs, together with other prognostic parameters (N=654)**

|  | **Univariate** | | **Multivariate** | |
| --- | --- | --- | --- | --- |
| **Parameter** | **HR (95% CI)** | ***P*** | **HR (95% CI)** | ***P*** |
| **RDW** | | | | |
| Low (<14.15) | 1 (reference) |  |  |  |
| High (≥14.15) | 0.935 (0.721 - 1.213) | 0.615 |  |  |
| **NLR** | | | | |
| Low (<5.25) | 1 (reference) |  | 1 (reference) |  |
| High (≥5.25) | 1.457 (1.137 - 1.867) | 0.003 | 1.331 (1.035 - 1.712) | 0.026 |
| **PLR** | | | | |
| Low (<273.5) | 1 (reference) |  | 1 (reference) |  |
| High (≥273.5) | 1.776 (1.406 - 2.244) | <0.001 | 1.255 (0.976 - 1.614) | 0.077 |
| **MLR** | | | | |
| Low (<0.45) | 1 (reference) |  | 1 (reference) |  |
| High (≥0.45) | 1.565 (1.232 – 1.986) | <0.001 | 1.245 (0.972 - 1.595) | 0.083 |
| **Combined RDW+NLR** | | | | |
| RDW-low + NLR-  low | 1 (reference) |  | 1 (reference) |  |
| RDW-high or NLR-  high | 1.382 (1.082 - 1.765) | 0.010 | 1.210 (0.943 - 1.552) | 0.134 |
| RDW-high + NLR-  high | 1.098 (0.706 - 1.708) | 0.678 | 1.029 (0.658 - 1.607) | 0.901 |
| **Age at diagnosis, years** | | | | |
| <55 | 1 (reference) |  |  |  |
| 55-63 | 1.501 (1.100 - 2.047) | 0.010 |  |  |
| 63-72 | 1.313 (0.965 - 1.785) | 0.083 |  |  |
| ≥72 | 1.110 (0.781 - 1.576) | 0.561 |  |  |
| **Origin of cancer** | | | | |
| Ovary | 1 (reference) |  | **1 (reference)** |  |
| Fallopian tube | 1.174 (0.521 - 2.644) | 0.698 | **0.989 (0.436 - 2.244)** | **0.978** |
| Peritoneum | 1.683 (1.300 - 2.178) | <0.001 | **1.071 (0.818 - 1.402)** | **0.617** |
| **Stage** | | | | |
| I | 1 (reference) |  | **1 (reference)** |  |
| II | 2.859 (1.187 - 6.852) | 0.019 | **2.149 (0.879 - 5.254)** | **0.094** |
| III | 9.592 (5.077 - 18.121) | <0.001 | **4.092 (2.017 - 8.304)** | **<0.001** |
| IV | 9.660 (4.883 - 19.112) | <0.001 | **3.998 (1.875 - 8.525)** | **<0.001** |
| **Histology** | | | | |
| High-grade serous | 1 (reference) |  | **1 (reference)** |  |
| Low-grade serous | 0.252 (0.035 - 1.797) | 0.169 | **1.005 (0.057 - 17.801)** | **0.997** |
| Endometrioid | 0.211 (0.120 - 0.368) | 0.000 | **0.464 (0.239 - 0.901)** | **0.023** |
| Clear cell | 0.424 (0.232 - 0.777) | 0.005 | **0.879 (0.433 - 1.786)** | **0.721** |
| Mucinous | 0.000 (0.000 - ~) | 0.916 | **0.000 (0.081 - ~)** | **0.929** |
| **Grade** | | | | |
| 1 | 1 (reference) |  | **1 (reference)** |  |
| 2 | 4.122 (0.942 - 18.025) | 0.600 | **1.531 (0.188 - 12.466)** | **0.690** |
| 3 | 12.990 (3.229 - 52.260) | <0.001 | **1.704 (0.209 - 13.874)** | **0.618** |
| **Residual disease** | | | | |
| No macroscopic disease | 1 (reference) |  | **1 (reference)** |  |
| Macroscopic disease <1 cm | 2.212 (1.721 - 2.843) | <0.001 | **1.381 (1.056 - 1.806)** | **0.018** |
| Macroscopic disease >1 cm | 2.998 (1.994 - 4.506) | <0.001 | **1.687 (1.096 - 2.596)** | **0.017** |

Univariate and multivariate analysis performed using Cox proportional hazards models. RDW, NLR, PLR, MLR, and combined RDW+NLR were adjusted separately in models that included origin of cancer, stage, histology, grade and residual disease. Preoperative CA125 level and ascites at surgery were excluded because of missing values (16.3% and 18.3%, respectively). Results from multivariate model which included NLR are indicated in bold (RDW and age were not included in multivariate analysis for they showed no significance in univariate analysis). Abbreviations: RDW = red blood cell distribution width; NLR = neutrophil-to-lymphocyte ratio; PLR = platelet-to-lymphocyte ratio; MLR = monocyte -to-lymphocyte ratio; HR = hazard ratio; CI = confidence interval.
